# Validation of a Greek version of the oral health impact profile (OHIP-14) for use among adults

**DOI:** 10.1186/1477-7525-10-7

**Published:** 2012-01-14

**Authors:** Vassilia Papagiannopoulou, Constantine J Oulis, William Papaioannou, George Antonogeorgos, John Yfantopoulos

**Affiliations:** 1 School of Law, Economic and Political Sciences, University of Athens, Greece; 2 School of Dentistry, University of Athens, Greece; 3 Department of Nutrition and Dietetics, Harokopio University, Greece

## Abstract

**Background:**

To test the validity of the short form of the Oral Health Impact Profile (OHIP-14) for use among adults in Greece.

**Methods:**

The original English version of the OHIP-14 was translated using the forward-backward technique, pilot-tested, and then applied to 211 adults aged 35 years and above. The questionnaire was filled out during face-to-face interviews conducted by one dentist, while individuals were asked to undergo a clinical examination. The internal consistency of the questionnaire was evaluated using Cronbach's alpha (α) coefficient and inter-item and item-total correlations. Discriminant and convergent validities were assessed.

**Results:**

Cronbach's α was estimated to be 0.90. Inter-item correlations coefficients ranged from 0.10 to 0.83, while item-total correlations coefficients from 0.44 to 0.76. Significant associations were found between OHIP-14 and the decayed, missing and filled teeth (DMFT) and oral hygiene, supporting the ability of the questionnaire to discriminate between individuals with and without impacts. The OHIP-14 total score was highly associated with self-perceived oral health status (r_s _= 0.57; p = 0.01), as well as with self-assessment of oral satisfaction (r_s _= 0.55;p = 0.01). Similar results were observed by investigating the relationship between the latter questions and each domain score as well as in various sub-groups analyses.

**Conclusions:**

The OHIP-14 is a reliable and valid questionnaire for the assessment of OHRQoL among adults in Greece.

## Background

Health-Related Quality of Life (HRQoL) is a multi-dimensional concept, which refers to patients' physical, psychological and social well-being, and is widely recognised for the assessment of healthcare outcomes. A factor, however, that can significantly impact on the construct of HRQoL is the oral health of the individual. Oral Health-Related Quality of Life (OHRQoL) measures have been widely used in the evaluation of oral health needs and combined with clinical indicators in order to better identify not only patients' symptoms due to oral diseases but also patients' ability to perform their daily activities [1,2]. One of the most widely known OHRQoL instruments is the short form of the Oral Health Impact Profile consisting of 14 items (OHIP-14), which is derived from the original 49-item version developed by Slade and Spenser [3], for the measurement of disability and discomfort due to oral conditions. This instrument has been translated and validated in many languages in different regions of the world [4-13]. In Greece, so far and to our knowledge only one OHRQoL measure has been tested for validity in an adult population: the Oral Impacts on Daily Performance (OIDP) [14], while no such attempt regarding the OHIP-14 has been undertaken. The latter shows slightly better correlation with clinical measures of oral health status [15] and appears to be more useful to discriminate between groups with and without impacts in population surveys [16].

The objective of this study was to translate the original English version of the OHIP-14 into Greek, and test its validity and reliability for use among Greek adults.

## Methods

A Greek version of the OHIP-14 was developed and its psychometric properties were tested in 2 stages: 1) a linguistic translation of the original OHIP-14 into Greek and 2) completion of a main study to evaluate the construct's validity.

For the OHIP-14 to be translated, four independent translations were conducted: two forward and two backward translations. Following comparison of these two forward translations, to ensure the best interpretation of the original version, the preliminary Greek version of the OHIP-14 was generated. Afterwards, two independent bilingual individuals unfamiliar with the original version, whose first language was English, were asked to conduct the backward translations. The backward translations were then compared to the original English version to check the similarity of their structure. The final version of the Greek OHIP-14 was produced after minor modifications were made according the results of a pilot study. The participants consisted of a convenience sample of 20 adult patients undergoing a dental check-up in the University of Athens Dental School. Those presenting with acute dental problems or oral disease were excluded.

### Sample design

The study was conducted in the two metropolitan regions of Athens and Thessaloniki, where according to the latest Population Census (2001) 45% of the Greek population resides. The sample units were the same as for the National Pathfinder Survey [17] and consisted of: i) the examination clinics of the Dental Schools of Athens and Thessaloniki ii) dental clinics of the Social Insurance Fund of Greece (IKA) in Athens and Thessaloniki and iii) a selected professional corporation. These units were selected based on: a) the availability of an appropriate middle-age group of the population in one place, b) the possibility to perform a clinical examination along with completion of a questionnaire and 3) the population found in these units, in terms of their socio-demographic strata and oral health condition, were closer to the characteristics of the General population based on the findings of the National Pathfinder survey [17].

Thus, a consecutive sample of 211 healthy Greek individuals aged 35 yrs and above visiting the aforementioned sampling units for dental check-up or treatment were interviewed and clinically examined.

All subjects were acquainted with the purpose of the study, which was ethically approved by the Research Committee of the School of Dentistry. Out of 250 approached individuals, 211 agreed to participate in the study (a response rate of 84%), all of who provided informed consent.

A self-administrated questionnaire was designed and one dentist trained in OHRQoL terms conducted face-to-face interviews. Participants were asked to evaluate on a 5-point Likert scale (0 = never, 1 = hardly ever, 2 = occasionally, 3 = fairly often and 4 = very often) how frequently during the last year had experienced any of the problems assessed by the 14-item OHIP. Data regarding their socio-demographic profile were also recorded. Besides OHIP-14, the questionnaire also included items for the assessment of the different types of construct validity given the absence of a universally accepted "gold standard"(*i.e.*, self-perceived general and oral health status). After the completion of the questionnaire, all participants underwent a clinical examination. One experienced and calibrated dentist recorded the number of decayed, missing and filled teeth according to the BASCD criteria [18], as well as the Oral Hygiene Index, in accordance with which subjects' hygiene might be categorized into 3 sub-groups representing good (0-1.2), fair (1.3-3.0) or poor (3.0-6.0) oral hygiene respectively [19].

### Scoring Method and Data Analysis

The OHIP-14 score was calculated using the additive method. Statistical analysis was performed using the Statistical Package for Social Sciences (SPSS) v.19.

To assess the reliability of the OHIP-14, Cronbach's *α *coefficient was used. In addition, the impact on the alpha value by the removal of OHIP-14 items (alpha if item deleted) was evaluated, as well as inter-item and item-total correlations.

Two types of construct validity were used. First, discriminant validity was evaluated by examining the association between the OHIP-14 total score and participants' dental status as assessed by the clinical examination. Mann-Whitney or Kruskal-Wallis test was used to assess the significance of differences between groups. Secondly, the convergent validity of the OHIP-14 was assessed by investigating the association (Spearman's correlation coefficient (r_s_)) among OHIP-14 total score and each domain score with self-perceived oral health status (good, fair and poor), and self-assessment of oral satisfaction (satisfied, dissatisfied). Participants' perception about their general health status was also associated with the OHIP-14 total score. Furthermore, sub-group analyses was performed in order to evaluate possible differences according to the region, Athens or Thessaloniki or the sampling cluster units - University dental clinics or social insurance dental clinics (selected corporation professionals due to the small participants number (n = 6) were merged with the social insurance sub-group). Finally, in order to further validate these results regression analysis was pursued where the dependent variable was the OHIP-14 total score and the independent variables were the subscales of OHIP-14.

All descriptive and model based results were derived by taking into account the hierarchical nature of the sample (i.e. individuals clustered in dental clinics) while in the multivariate model the adjusting factors for gender, residence, education, and occupational status were taken into account in order to minimize potential confounding effects.

## Results

In total, 211 individuals participated in the study with a mean age of 53.3 years (S.D. 15.4), with the majority being females (52.9%), employed (51.1%), with secondary education (57%) and living in the capital of Athens (53.8%). The mean value of the decayed, missing and filled teeth (DMFT) index was 16.8 (S.D. 7.7), with 1.0 (S.D. 1.9) being decayed, 10.8 (S.D. 10.1) missing and 5.0 (S.D. 4.9) filled teeth. Finally, the mean number of present teeth (including abutment teeth) was estimated to be 21.1 (S.D. 10.1), while 48.5% of the sample had good, 36.3% fair, and 15.2% poor oral hygiene.

In order to examine the possible design effect of our study, we stratified our analysis according to the sampling region (Athens or Thessaloniki) and to the sampling cluster units (university or social insurance dental clinics). The number or defined clusters were as follows: from Athens, one University dental clinic with 23 participants, and 4 Social Insurance dental clinics with 84 participants (6 participants came from a professional corporation but due to the small size we merged them with one of the Social Insurance dental clinics). From Thessaloniki, 49 participants came from the University dental clinic and 49 participants came from two Social Insurance dental clinics.

By analyzing participants' OHRQoL data, a high level of oral health impacts was observed. More specifically, the mean total score of the OHIP-14 was 14.9 (S.D. 10.0), with the most affected sub-scales being those of Functional Limitation and Psychological Discomfort both with a mean value of 2.9 (S.D. 2.0). The Physical pain sub-scale was also highly rated (mean 2.6, S.D. 2.0), followed by the Handicap (mean 2.2, S.D. 1.5) and Physical Disability (mean 2.0, S.D. 1.9) sub-scales. Finally, the least affected sub-scales were Psychological and Social Disability with mean values 1.4 (S.D. 1.8) and 1.0 (S.D. 1.4) respectively.

### Reliability

The Cronbach's alpha value of the OHIP-14 was estimated to be 0.90, representing an excellent internal consistency. The removal of one item at a time resulted in lower alpha values than the original one, supporting the inclusion of all items. By analyzing the matrix of inter-item correlations (Table 1), a positive correlation between all items was found. Finally, as it is shown in Table 2 the item-total correlations coefficients were above 0.20, which is recommended as the minimum value for including an item in a scale [20]. Sub-group analyses confirmed the stability of the above findings regardless metropolitan region or dental clinic variations (Table 3).

**Table 1:** Reliability analysis based on the OHIP inter-item correlation.

	1	2	3	4	5	6	7	8	9	10	11	12	13	14
**1**	1.00													
**2**	0.38	1.00												
**3**	0.80	0.46	1.00											
**4**	0.34	0.41	0.35	1.00										
**5**	0.50	0.46	0.53	0.41	1.00									
**6**	0.37	0.35	0.43	0.32	0.38	1.00								
**7**	0.66	0.40	0.68	0.48	0.61	0.49	1.00							
**8**	0.26	0.23	0.28	0.23	0.23	0.74	0.40	1.00						
**9**	0.39	0.26	0.41	0.36	0.31	0.36	0.52	0.43	1.00					
**10**	0.38	0.35	0.41	0.34	0.34	0.35	0.46	0.38	0.83	1.00				
**11**	0.28	0.19	0.34	0.16*	0.21	0.57	0.41	0.51	0.45	0.47	1.00			
**12**	0.34	0.31	0.35	0.25	0.25	0.44	0.39	0.39	0.52	0.59	0.60	1.00		
**13**	0.44	0.29	0.45	0.28	0.43	0.20	0.43	0.10	0.30	0.3	0.21	0.18	1.00	
**14**	0.32	0.33	0.35	0.36	0.27	0.69	0.41	0.63	0.48	0.51	0.62	0.50	0.14*	1.00

All Correlations are significant at the 0.01 level (2-tailed) except for *two at the 0.05 level (2-tailed).

**Table 2:** Reliability analysis based on the corrected item-total correlation and Cronbach's alpha coefficient if item deleted.

Impact Item	Corrected Item - Total Correlation	Cronbach's Alpha if Item Deleted
**1 **Difficult to pronounce words	0.65	0.89
**2 **Worsened taste	0.52	0.89
**3 **Pain	0.70	0.89
**4 **Uncomfortable to eat	0.50	0.89
**5 **Self-conscious	0.58	0.89
**6 **Feel tensed	0.65	0.89
**7 **Diet unsatisfactory	0.76	0.88
**8 **Interrupted meals	0.54	0.89
**9 **Difficult to relax	0.64	0.89
**10 **Embarrassed	0.64	0.89
**11 **Irritable	0.56	0.89
**12 **Difficult to do jobs	0.57	0.89
**13 **Life less satisfying	0.44	0.89
**14 **Totally unable to function	0.64	0.89

**Table 3:** Reliability analysis based on the corrected item-total correlation and Cronbach's alpha coefficient if item deleted according to the sampling region and the sampling cluster units of the study.

Impact Item	Athens		Thessaloniki		University Dental Clinic		Social Insurance Dental Clinic	
	
	Corrected Item - Total Correlation	Cronbach's Alpha if Item Deleted	Corrected Item - Total Correlation	Cronbach's Alpha if Item Deleted	Corrected Item - Total Correlation	Cronbach's Alpha if Item Deleted	Corrected Item - Total Correlation	Cronbach's Alpha if Item Deleted
1. Difficult to pronounce words	0.64	0.88	0.66	0.85	0.66	0.91	0.64	0.88
2. Worsened taste	0.50	0.88	0.36	0.87	0.54	0.92	0.51	0.88
3. Pain	0.73	0.88	0.68	0.85	0.77	0.91	0.66	0.88
4. Uncomfortable to eat	0.43	0.88	0.34	0.87	0.63	0.92	0.41	0.89
5. Self-conscious	0.48	0.88	0.60	0.85	0.65	0.92	0.54	0.88
6. Feel tensed	0.61	0.88	0.70	0.85	0.74	0.91	0.60	0.88
7. Diet unsatisfactory	0.72	0.88	0.72	0.85	0.81	0.91	0.73	0.87
8. Interrupted meals	0.55	0.88	0.51	0.86	0.58	0.92	0.52	0.88
9. Difficult to relax	0.69	0.88	0.43	0.86	0.69	0.91	0.60	0.88
10. Embarrassed	0.67	0.88	0.42	0.86	0.69	0.91	0.62	0.88
11. Irritable	0.57	0.88	0.46	0.86	0.58	0.92	0.56	0.88
12. Difficult to do jobs	0.61	0.88	0.32	0.87	0.46	0.92	0.65	0.88
13. Life less satisfying	0.16	0.89	0.51	0.86	0.54	0.92	0.39	0.89
14. Totally unable to function	0.62	0.88	0.57	0.86	0.73	0.91	0.58	0.88

### Construct Validity

In Table 4 the results of the assessment of discriminant validity are presented. As hypothesised, participants' with a high OHIP-14 score presented a higher number of decayed and missing teeth and a lower number of natural and filled teeth. All differences were statistically significant. The ability of the OHIP-14 to discriminate between groups was also confirmed by evaluating its association with the Oral Hygiene Index. Participants with poor oral hygiene had higher OHIP scores than those with fair or good oral hygiene (p < 0.01).

**Table 4:** Discriminant validity of the OHIP-14 based on the clinical status of the subjects.

Variable (number of cases)	OHIP-14 Mean (S.D.*)	Test
**Number of teeth:**		
< 25 teeth	17.3 (11.3)	Mann-Whitney
≥ 25 teeth	12.4 (7.6)	P < 0.01

**Number of decayed teeth:**		
Dt < 2	13.7 (9.4)	Mann-Whitney
Dt ≥ 2	18.2 (10.7)	P < 0.05

**Number of missing teeth:**		
Mt < 8	12.8 (8.0)	Mann-Whitney
Mt ≥ 8	17.7 (11.5)	P < 0.01

**Number of filled teeth:**		
Ft < 6	16.3 (10.9)	Mann-Whitney
Ft ≥ 6	12.8 (7.7)	P < 0.05

**Oral Hygiene Index (OHI-s) distribution**		
OHΙ-s = 0.0-1.2 (good oral hygiene)	11.9 (8.5)	Kruskall-Wallis
OHΙ-s = 1.3-3.0 (fair oral hygiene)	16.0 (9.6)	P < 0.01
OHΙ-s = 3.1-6.0 (poor oral hygiene)	18.8 (10.7)	

S.D. = Standard Deviation

With regard to the convergent validity of the OHIP-14, it was estimated that as the participants' perceived oral health status changed from poor to good both the mean OHIP total score and the subscales scores increased (Table 5). All the Spearman's rank correlation coefficients were positive and statistically significant. No significant differences in our results were noted when the effect of the sampling region of the study (Athens or Thessaloniki) or the sampling cluster units (university or social insurance dental clinics) was accounted for (Table 6). Moreover, similar results were observed by associating the OHIP-14 total score with participants' perception of their general health status. Those who evaluated their general health status being good, had a lower OHIP-14 score than those reporting fair or poor general health status, whilst the Spearman's rank correlation coefficient was 0.22, significant at the 0.01 level.

**Table 5:** Convergent validity of the OHIP-14: Mean scores and Spearman's rank Correlation coefficients among the OHIP-14 and subscale scores and self-perceived Oral health status.

	Self-perceived oralhealth status				
**Subscales**	**All subjects n = 210** ***Mean (S.D.)***	**Good** **n = 103** ***Mean (S.D.)***	**Fair** **n = 83** ***Mean (S.D.)***	**Poor** **n = 24** ***Mean (S.D.)***	**r_s_**

**Functional limitation**	2.9 (2.0)	2.1 (1.7)	3.1 (1.6)	5.5 (1.7)	0.45*
**Physical pain**	2.6 (2.0)	1.9 (1.6)	2.9 (1.6)	4.5 (2.3)	0.39*
**Psychological discomfort**	2.9 (2.0)	2.0 (1.6)	3.3 (1.9)	4.8 (1.7)	0.47*
**Physical disability**	1.4 (1.8)	0.8 (1.5)	1.5 (1.6)	3.5 (2.4)	0.41*
**Psychological disability**	2.0 (1.9)	1.0 (1.2)	2.5 (1.9)	4.5 (1.7)	0.57*
**Social disability**	1.0 (1.4)	0.5 (1.1)	1.2 (1.3)	2.5 (2.3)	0.39*
**Handicap**	2.2 (1.5)	1.6 (1.3)	2.5 (1.3)	3.6 (1.4)	0.45*
**OHIP-14**	14.9 (10.0)	9.8 (7.4)	17.2 (7.6)	29.0 (10.1)	0.57*

*Correlation is significant at the 0.01 level (2-tailed).

**Table 6:** Convergent validity of the OHIP-14: Mean scores and Spearman's rank Correlation coefficients among the OHIP-14 and subscale scores and self-perceived Oral health status according to the sampling region and the sampling cluster units of the study.

	Self-perceived oral health status				
**Subscales**	**All subjects n = 210** ***Mean (S.D.)***	**Good** **n = 103** ***Mean (S.D.)***	**Fair** **n = 83** ***Mean (S.D.)***	**Poor** **n = 24** ***Mean (S.D.)***	**r_s_**

***Athens***					

**Functional limitation**	3.4 (2.0)	2.5 (1.6)	3.4 (1.7)	5.7 (1.9)	0.37*
**Physical pain**	3.2 (1.8)	2.5 (1.4)	3.3 (1.5)	5.1 (2.2)	0.27*
**Psychological discomfort**	4.4 (2.3)	3.3 (1.6)	4.4 (1.9)	7.2 (2.2)	0.45*
**Physical disability**	1.8 (1.7)	1.0 (1.4)	1.8 (1.3)	3.9 (2.1)	0.40*
**Psychological disability**	2.5 (1.8)	1.4 (1.2)	2.6 (1.5)	4.8 (1.4)	0.51*
**Social disability**	1.4 (1.6)	0.8 (1.2)	1.4 (1.2)	3.0 (2.3)	0.35*
**Handicap**	1.9 (0.8)	1.9 (0.9)	2.0 (0.8)	2.0 (1.0)	0.02
**OHIP-14**	18.8 (9.5)	13.3 (6.5)	18.8 (6.6)	31.6 (9.2)	0.47*

***Thessaloniki***					

**Functional limitation**	2.3 (1.8)	1.7 (1.7)	2.6 (1.5)	4.8 (0.4)	0.41*
**Physical pain**	1.9 (1.7)	1.5 (1.6)	2.4 (1.8)	2.6 (1.5)	0.35*
**Psychological discomfort**	2.4 (2.3)	1.7 (2.0)	3.4 (2.4)	4.2 (2.6)	0.43*
**Physical disability**	0.8 (1.2)	0.4 (0.9)	1.4 (1.4)	1.8 (1.1)	0.43*
**Psychological disability**	1.3 (1.9)	0.5 (0.9)	2.4 (2.3)	3.4 (2.5)	0.40*
**Social disability**	0.5 (1.0)	0.3 (0.8)	0.9 (1.3)	0.6 (0.9)	0.34*
**Handicap**	1.2 (1.1)	0.9 (1.0)	1.5 (1.1)	1.6 (0.9)	0.36
**OHIP-14**	10.2 (8.5)	6.9 (6.9)	14.6 (8.4)	19.0 (7.0)	0.52*

***University Dental Clinic***					

**Functional limitation**	2.9 (2.2)	1.8 (1.8)	3.0 (1.9)	5.1 (2.1)	0.42*
**Physical pain**	2.5 (2.3)	1.3 (1.6)	3.0 (2.2)	4.7 (2.5)	0.51*
**Psychological discomfort**	3.4 (2.8)	1.8 (2.1)	4.0 (2.1)	7.1 (2.7)	0.63*
**Physical disability**	1.4 (1.8)	0.5 (1.3)	1.8 (1.6)	3.6 (2.1)	0.53*
**Psychological disability**	2.1 (2.0)	0.8 (1.1)	2.8 (1.7)	4.8 (1.5)	0.67*
**Social disability**	1.0 (1.4)	0.6 (1.2)	1.0 (1.1)	2.7 (1.8)	0.42*
**Handicap**	1.4 (1.0)	1.0 (1.1)	1.7 (1.0)	2.0 (0.7)	0.37*
**OHIP-14**	14.7 (11.3)	7.9 (8.1)	17.4 (7.7)	30.0 (9.9)	0.66*

***Social Insurance Dental Clinic***					

**Functional limitation**	2.9 (1.9)	2.1 (1.7)	3.1 (1.6)	5.8 (1.5)	0.47*
**Physical pain**	2.7 (1.7)	2.2 (1.6)	2.8 (1.3)	4.4 (2.2)	0.32*
**Psychological discomfort**	3.6 (2.3)	2.7 (1.9)	4.0 (2.2)	6.1 (2.5)	0.44*
**Physical disability**	1.3 (1.5)	0.7 (1.2)	1.5 (1.2)	3.4 (2.2)	0.44*
**Psychological disability**	1.9 (1.9)	1.0 (1.2)	2.4 (1.9)	4.3 (1.9)	0.51*
**Social disability**	1.0 (1.5)	0.4 (0.9)	1.3 (1.3)	2.4 (2.6)	0.38*
**Handicap**	1.7 (1.0)	1.5 (1.1)	1.9 (0.9)	0.9 (1.2)	0.15*
**OHIP-14**	15.0 (9.3)	10.7 (9.0)	17.1 (7.6)	28.3 (10.6)	0.52*

*Correlation is significant at the 0.01 level (2-tailed).

The convergent validity of the OHIP-14 was also supported by examining the relationship among the OHIP-14 total score and each domain score with self-assessment of oral satisfaction. According to Table 7 in which the findings of this association are summarized, participants who were not satisfied with their oral health status had statistically significant higher OHIP-14 or sub-scale scores compared to those who were. Similar patterns of results were observed regarding the convergence validity of the OHIP-14 with the participants' perceived oral satisfaction when differences in the sampling region or in the place where the study conducted were assessed by sub-group analyses except for the non-significant association for the Handicap sub-scale in the Athens sample (Table 8).

**Table 7:** Convergent validity of the OHIP-14: Mean scores and Spearman's rank correlation coefficients among the OHIP-14 and subscale scores and self-assessment of oral satisfaction.

	Self-assessment of oral satisfaction			
**Subscales**	**All subjects** **n = 202** ***Mean (S.D.)***	**Satisfied** **n = 127** ***Mean (S.D.)***	**Dissatisfied** **n = 75** ***Mean (S.D.)***	**r_s_**

**Functional limitation**	2.9 (2.0)	2.2 (1.7)	4.0 (2.0)	0.43*
**Physical pain**	2.6 (2.0)	2.1 (1.7)	3.5 (1.9)	0.36*
**Psychological discomfort**	2.9 (2.0)	2.1 (1.7)	4.1 (1.8)	0.47*
**Physical disability**	2.0 (1.9)	1.2 (1.4)	3.4 (2.0)	0.53*
**Psychological disability**	1.4 (1.8)	0.9 (1.5)	2.3 (2.0)	0.40*
**Social disability**	1.0 (1.4)	0.5 (1.0)	1.7 (1.7)	0.40*
**Handicap**	2.2 (1.5)	1.6 (1.3)	3.1 (1.3)	0.48*
**OHIP-14**	14.9 (10.0)	10.6 (7.5)	22.3 (9.4)	0.55*

*Correlation is significant at the 0.01 level (2-tailed).

**Table 8:** Convergent validity of the OHIP-14: Mean scores and Spearman's rank correlation coefficients among the OHIP-14 and subscale scores and self-assessment of oral satisfaction by the sampling region and the sampling cluster units of the study.

	Self-assessment of oral satisfaction			
***Athens***				

**Subscales**	**All subjects** **n = 202** ***Mean (S.D.)***	**Satisfied** **n = 127** ***Mean (S.D.)***	**Dissatisfied** **n = 75** ***Mean (S.D.)***	**r_s_**

**Functional limitation**	3.4 (2.0)	2.7 (1.6)	4.2 (2.1)	0.37*
**Physical pain**	3.2 (1.8)	2.7 (1.5)	3.4 (1.7)	0.27*
**Psychological discomfort**	4.4 (2.3)	3.4 (1.7)	5.4 (2.4)	0.45*
**Physical disability**	1.8 (1.7)	1.1 (1.4)	2.6 (1.9)	0.40*
**Psychological disability**	2.5 (1.8)	1.6 (1.3)	3.4 (1.7)	0.51*
**Social disability**	1.4 (1.6)	0.9 (1.2)	2.0 (1.8)	0.35*
**Handicap**	1.9 (0.8)	1.9 (0.9)	2.0 (0.9)	0.02
**OHIP-14**	18.8 (9.5)	14.3 (6.8)	23.4 (9.7)	0.47*

***Thessaloniki***				

**Functional limitation**	2.3 (1.8)	1.7 (1.6)	3.7 (1.6)	0.41*
**Physical pain**	1.9 (1.7)	1.5 (1.6)	2.9 (1.5)	0.35*
**Psychological discomfort**	2.4 (2.3)	1.8 (2.0)	4.3 (2.3)	0.43*
**Physical disability**	0.8 (1.2)	0.4 (0.9)	2.0 (1.4)	0.43*
**Psychological disability**	1.3 (1.9)	0.7 (1.2)	3.3 (2.6)	0.40*
**Social disability**	0.5 (1.0)	0.3 (0.8)	1.2 (1.4)	0.34*
**Handicap**	1.2 (1.1)	0.9 (1.0)	1.9 (1.0)	0.36*
**OHIP-14**	10.2 (8.5)	7.5 (6.6)	19.2 (8.1)	0.52*

***University Dental Clinic***				

**Functional limitation**	3.4 (2.0)	1.8 (1.7)	4.0 (2.2)	0.44*
**Physical pain**	3.2 (1.8)	1.5 (1.8)	3.8 (2.2)	0.52*
**Psychological discomfort**	4.4 (2.3)	1.8 (1.8)	5.6 (2.6)	0.66*
**Physical disability**	1.8 (1.7)	0.6 (1.2)	2.6 (2.0)	0.55*
**Psychological disability**	2.5 (1.8)	0.9 (1.1)	3.7 (2.0)	0.58*
**Social disability**	1.4 (1.6)	0.4 (1.0)	1.9 (1.6)	0.49*
**Handicap**	1.9 (0.8)	1.1 (1.0)	1.9 (0.9)	0.42*
**OHIP-14**	18.8 (9.5)	8.1 (6.9)	23.6 (10.0)	0.67*

***Social Insurance Dental Clinic***				

**Functional limitation**	2.3 (1.8)	2.3 (1.6)	4.1 (1.9)	0.42*
**Physical pain**	1.9 (1.7)	2.3 (1.6)	3.4 (1.7)	0.27*
**Psychological discomfort**	2.4 (2.3)	2.9 (2.1)	4.9 (2.2)	0.42*
**Physical disability**	0.8 (1.2)	0.8 (1.2)	2.3 (1.7)	0.43*
**Psychological disability**	1.3 (1.9)	1.2 (1.4)	3.2 (2.0)	0.50*
**Social disability**	0.5 (1.0)	0.6 (1.1)	1.7 (1.8)	0.36*
**Handicap**	1.2 (1.1)	1.5 (1.1)	2.0 (0.9)	0.17*
**OHIP-14**	10.2 (8.5)	11.6 (7.5)	21.4 (9.1)	0.49*

*Correlation is significant at the 0.01 level (2-tailed).

When examining the validity of the OHIP-14 scale one should not restrict himself to simple bivariate relationships since some possible confounding factors related to age, sex, place of residence, education and occupation should be taken into consideration. In the present analysis multivariate techniques based on regression analysis were adopted in order to avoid confounding bias. The dependent variable was the OHIP-14 total score and the independent variables were the subscales of OHIP-14 including the control variables for age, sex, place of residence, education and occupational status. From the seven subscales of OHIP-14, the dimension Handicap was selected to be excluded because of having the lowest correlation coefficient (r = 0.545) with OHIP-14 total score. The remaining six dimensions presented much higher correlation coefficients with OHIP-14 total score ranging from (r = 0.672) for Social Disability to (r = 0.867) for Physical Discomfort.

The results of the multivariate analysis with the best possible fit (after examining several models) are shown in Table 9. The value of adjusted R^2 ^(coefficient of determination) reveals that the estimated model explains approximately 99% of the variance of the dependent variable (OHIP Total Score). All the remaining dimensions of OHIP-14 (Physical Disability, Social Disability, Physical Discomfort, Physical Pain, Functional Limitation and Psychological Discomfort) are statistically significant at the p < 0.001. In addition the control variables of residence appears to be significant at the p < 0.005 level.

**Table 9:** Multivariate Regression Analysis between OHIP-14 Total Score (Dependent variable) and the subscales of OHIP-14 (Independent variables) adjusting for Gender, Residence, Education, and Occupational status.

Dependent Variable Add OHIP-14	Unstandardized Coefficients		t-ratios	Confidence Intervals		P-value
			
Independent variables	B	Std. Error		Lower	Upper	
Physical disability	0.906	0.078	11.647	0.752	1.060	< 0.001
Social disability	0.955	0.071	13.502	0.815	1.095	< 0.001
Physical discomfort	0.999	0.073	13.640	0.854	1.144	< 0.001
Physical pain	1.141	0.064	17.771	1.014	1.268	< 0.001
Functional limitaion	1.119	0.059	19.093	1.003	1.235	< 0.001
Psychological discomfort	1.067	0.062	17.073	0.943	1.190	< 0.001
Oral Health	0.214	0.267	0.804	-0.313	0.742	0.423
Gender	0.111	0.157	0.704	-0.200	0.422	0.483
Residence	-0.509	0.180	-2.820	-0.865	-0.152	0.006
Educational level	-0.088	0.066	-1.319	-0.219	0.044	0.190
Occupational status	0.031	0.058	0.544	-0.083	0.146	0.587

(R^2 = ^0.997. Adjusted R^2 ^= 0.993)

## Discussion

This study aimed to generate and evaluate the Greek version of the OHIP-14, in terms of validity and reliability, for use among adult population. To this effect, the original English version of the OHIP-14 was translated using the forward-backward technique, pilot-tested in a convenience group of adults and then applied to a sample of the Greek population having approximately the same socio-demographic and oral health conditions, in order for its validity and reliability to be tested. A mean DMFT of 16.8 (S.D. 7.7) of this population (with a mean age of 53.3 years) is between the value of 14.06 and 20.63 found in the National pathfinder survey for the 34-44 and the 65-74-year-old Greek adults respectively [17]. The findings of our study, which is the first using the OHIP-14 in Greece confirm that the OHIP-14 is a reliable and valid instrument for the measurement of OHRQoL among adults in Greece.

The internal consistency of the Greek OHIP-14 was found to be excellent, with the Cronbach's alpha coefficient greatly exceeding the minimum recommended value of 0.7 [16,21]. Moreover, the Cronbach's alpha coefficient reported in our study was slightly better than those reported by Slade [21] in the original English version, as well as than those observed by Montero-Martin *et al *[12] who tested the reliability of the OHIP-14 among adult population in Spain, but the same with that found for Swedish adults [22]. In addition, lower alpha values were observed by the omission of any of the 14 items, which provides further evidence about the internal consistency of the present version of the OHIP-14.

The substantial internal consistency of the instrument was also supported by the findings regarding inter-item and item-total correlations. Specifically, all the inter-item correlations were positive, and none was high enough for any item to be redundant, while the item-total correlations coefficients were above the recommended threshold for including an item in a scale. Similar results have been observed in the Spanish [12] and the Sinhalese version [23] of the OHIP-14, both evaluating the reliability of the instrument among adults. As mentioned, the OHIP-14 proved to be a valid measure for assessing OHRQoL among Greek adults. This was firstly confirmed by its ability to discriminate between groups with different oral health status, which was objectively assessed by clinical measures. It was found that the more frequent the presence of oral conditions such as tooth loss or dental caries the greater the impact on individuals' OHRQoL. Indeed, it was found in the present study that the presence of less than 25 teeth negatively affects the OHRQoL measures (p < 0.01) in adults, confirming a previous study in the UK [24].

Moreover, a statistically significant association was found between questions aiming to subjectively evaluate individuals' oral health status (such as self-perceived oral health status and self-assessment of oral satisfaction) and OHIP-14 scores. This provided further evidence for the instrument's construct validity, since it was shown that the higher the OHIP-14 total and the subscale scores, the more diminished the OHRQoL, and thus the poorer the perceived oral health status and satisfaction. Similar findings were presented for the UK by McGrath and Bedi [24]. Finally, the fact that the Spearman's rank coefficient for the association between the OHIP-14 total score and self-perceived oral health status was higher than those for the association between the OHIP-14 total score and self-perceived general health status (0.57 vs. 0.22), is not only in accordance with previous findings in the literature [12,25], but also inarguably supports the specificity of the index.

Concerning the calculation of sample size, a confidence interval of +/-3% is considered desirable in high quality surveys. By keeping confidence intervals narrow we increase the expectation of making accurate statistical inferences, but a sample size of more than 1,300 subjects would be necessary to satisfy this principle. However, for validation studies this large number is impractical. In the current study the sample size of 211 individuals gives us a confidence interval of 6.7%. Indeed, in the literature similar studies have used similar sample sizes or ever smaller [4,7,9,11,12] whereas other fewer studies [5,8,23] in larger populations are based on larger sample sizes to validate the Oral Health Impact Profile (OHIP-14).

### Strengths and limitations of the study

An important strength of this study is that the selected sample was from the two largest cities of Athens and Thessaloniki, in which the same sample units as for the National Pathfinder survey [17] were utilized. This ensured that the current sample had sociodemographic and oral health conditions similar to those found in the survey for adult Greeks. The second is that the same person who undertook the interview was also a calibrated dentist. Therefore it was possible to combine quality of life subjective data with objective assessment of oral health based on clinical examination.

This study has several limitations. First the generalizability of quality of life findings might be limited to adult population of 35 years and above. Second, due to absence of "gold standards" in Greece, comparisons are not drawn with other quality of life instruments.

## Conclusions

The present findings indicate that the OHIP-14 is a reliable and valid measure to be used in studies focusing on the measurement of adults' OHRQoL in Greek adults, where although several HRQoL studies have been conducted for certain disease categories [26,27] little is known on whether the common oral health problems have an impact on OHRQoL of the individuals of the general population.

## Competing interests

The authors declare that they have no competing interests.

## Authors' contributions

**CJO **conceived the study, co-organised the epidemiological oral survey, discussed the quality of life section and corrected the final draft. **PV **drafted the manuscript organised the data selection and performed the validity and reliability statistical analysis. **WP **participated in drafting the paper and discussing the relevant literature in the OHRQoL. **AG **contributed to the statistical analysis, **YJ **coordinated the original study, designed the sampling process, examined the psychometric tests and corrected the final version.

All authors read and approved the final manuscript.
